# The RUsick2 Foodborne Disease Forum for Syndromic Surveillance

**DOI:** 10.3201/eid1003.030358

**Published:** 2004-03

**Authors:** Holly Wethington, Paul Bartlett

**Affiliations:** * National Food Safety and Toxicology Center, Michigan State University, East Lansing, Michigan, USA

**Keywords:** food poisoning, surveillance, gastrointestinal diseases, questionnaires

## Abstract

The RUsick2 Foodborne Disease Forum at the National Food Safety and Toxicology Center increased reporting of foodborne diseases to more than four times the rate seen in the previous 2 years. Since November 2002, the Forum has allowed pilot-area residents with sudden-onset vomiting or diarrhea to share and compare information regarding what they ate and did before becoming sick. The purpose is to identify a common food source, perhaps resulting in identifying a cluster of persons who ate the same contaminated food item. Such information can assist health departments in detecting foodborne outbreaks while the possibility for intervention remains.

Foodborne infection is the cause of approximately 76 million gastrointestinal illnesses and approximately 5,000 deaths each year in the United States (*1*), but causes are rarely identified. Nationally, an estimated 1%–2% of cases are reported annually (*2*). Given that two or three reported cases are required to recognize and define a common source outbreak, many small- and moderate-sized outbreaks escape detection. Table 1 depicts the results of a binomial analysis of outbreaks of various sizes, given an assumed 2% reporting. For example, with 2% reporting, an outbreak of 75 cases would have a 44% chance of having two or more cases reported and a 19% chance of having three or more cases reported. Many outbreaks are never detected, and many reported cases seen as sporadic and isolated may in fact be part of small, undetected outbreaks.

**Table 1:** Simulated binomial data, assuming 2% of cases reported

Size of outbreak	2 or more reports	3 or more reports
25	9%	1%
50	26%	8%
75	44%	19%

Current laboratory-based surveillance will likely not substantially increase the percentage of routine gastroenteritis cases that provide samples for culturing. Health insurance organizations are not expected to increase the numbers of fecal samples submitted and cultured for uncomplicated cases of gastroenteritis, since culture results do not usually influence the medical management of individual cases.

A second problem is the time delay inherent in current laboratory-based passive surveillance. On the basis of a 2000 survey of reported cases of foodborne illness, 263 Michigan hospital laboratories (response rate 91%) averaged a delay of 12.3 days between specimen collection and serotyping. In addition, a mean delay of 35 days occurred between symptom onset and completion of the case investigation form by the local health department (Michigan Department of Community Health, unpub. data). Given the short duration of most foodborne outbreaks, health department investigations are often a matter of documenting past events, with no real opportunity to quickly identify and remove contaminated food items to prevent further exposure.

A third constraint of current surveillance is that it is based almost entirely on paper forms or individual telephone reports to local health departments. This system can manage sporadic cases and small outbreaks, but larger outbreaks would quickly overrun the capacity of most local health departments. The inability to adequately investigate large outbreaks is especially important given the potential for intentional contamination of food supplies as an act of bioterrorism or biowarfare.

Examining these three limitations evolved into a plan to implement a syndromic surveillance forum in which clusters of foodborne disease could be quickly identified for further investigation. This system would act as a method to augment existing laboratory-based surveillance and would identify clusters of persons with suspected foodborne disease that warrant further investigation by health departments.

## The RUsick2 Forum

### Data Input

**Figure 1:**
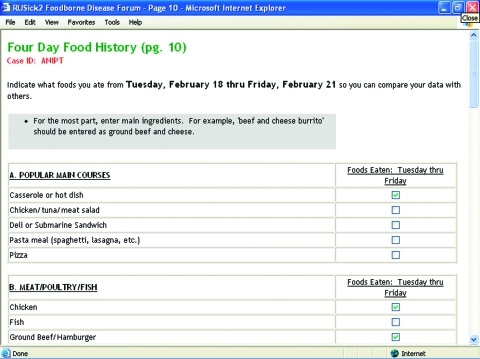
Abbreviated food history data entry screen.

A Web site (www.RUsick2.msu.edu) was developed to record information on symptoms, time of illness onset, a 4-day preillness food history, food sources, and other information regarding nonfoodborne sources of common gastrointestinal illness. Visitors can potentially view 22 screens, most of which are data input screens with a few displaying other visitors’ data (no personal identifiers are viewed by RUsick2 visitors). The Forum allows visitors to return multiple times to modify their data if they recall more about what food they consumed and where they purchased it. The Centers for Disease Control and Prevention (CDC) Standard Foodborne Questionnaire and other foodborne questionnaires were emulated in creating the data input screens (*3*). The food list currently contains 54 food items, divided into the following categories: popular main courses, meats/poultry/fish, dairy and eggs, raw fruit, raw vegetables, prepared fruit or vegetables, salad items/side dishes, grains and starches, and beverages. Figure 1 displays an abbreviated version of this data entry screen.

A section concerning nonfood exposures was incorporated to gather information regarding exposure to animals, sick persons, patients in a healthcare setting, commercial food preparation, young children, private well water, and swimming (lake/river or swimming pool). The Forum is unlike most Web-based forums in that it is structured and does not allow narrative testimony. As with written or telephone reporting, persons generally have a difficult time remembering what they consumed during the several days before becoming ill. Computer technology does not enhance memory, but it allows the reporter to recall data at his own pace and return to the Web site to add or modify data after consulting friends, family, calendars, checkbooks, and credit-card records.

A follow-up survey is being conducted of all visitors to the Web site. Virtually all modifications to the program instituted after November 2002 shortened and simplified the program. We intend to continue modifying the program to meet the requests of RUsick2 visitors. Moreover, focus groups are planned for the future to further increase usability.

## Information Retrieval

As the visitor proceeds through the program entering data regarding symptoms, food items eaten, and food sources, increasingly specific comparisons with other users’ data are available. The objective is to help each visitor determine what he might have in common with other persons, including symptoms, time of illness onset, and consumption of the same food item from the same food source.

**Figure 2:**
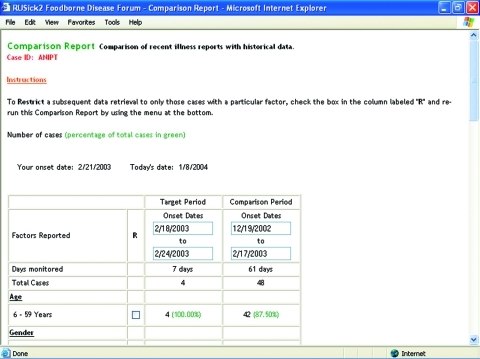
Comparison report displays each risk factor the visitor reported during his target period along with a comparison period.

The summary report is a univariate descriptive analysis showing the number and percentage of past visitors who reported the same risk factors (foods, food sources, nonfood exposures) as the current visitor, who can use the summary report to select individual reports for viewing. A comparison report analyzes the visitor’s risk factors during an adjustable “target” period of onset dates, compared to an adjustable historic “comparison” period. Subsequent retrievals can be restricted on the basis of risk factors, and data can be viewed as a case report or output in a format accessible by most spreadsheet, database, and statistical programs. Figure 2 shows an example of the comparison report.

Visitors may choose to enter their data and leave the investigation to the health departments to determine whether a cluster exists. They may also view a descriptive table of recent reports and see whether the source they originally suspected was mentioned by other Forum visitors. Persons who are satisfied that they are not part of an outbreak may leave the Forum without requesting further output. Those who see common exposures may pursue more sophisticated output. At any time in the process, Forum visitors can request the aid of their health department.

The Forum does not investigate outbreaks or replace the current systems used by local health departments. Rather, the Forum increases reporting of foodborne illness and makes identifying suspicious clusters that may warrant further investigation possible. By collecting numerous variables, the Forum delivers information on a large number of risk factors to local health departments to assist in an investigation.

A moderator views each report after it is entered and conducts appropriate follow-up. For example, if a report from outside the three-county pilot area is entered, the moderator will alert the proper health department that a report from their jurisdiction has been entered. Like most Web-based forums, the moderator also reviews data for reasonableness, profanity, or other infringements of posted Forum rules. Records can be excluded from analysis, and each health department may similarly reject records.

## Health Departments

RUsick2 visitors cannot retrieve other visitors’ personal identifiers, narrative testimony, or names of restaurants, stores, or other food sources. In contrast, health departments have access to all data fields for visitors who report being residents of their jurisdiction. Local health departments in the three-county pilot area around Lansing, Michigan, were involved in developing the Forum and have helped design methods by which local health departments can monitor the Forum. During the pilot phase, local health departments have had password access to the database to check reports from their residents. After expansion of the Forum, counties with less activity will be able to request automatic email notification from the Forum’s computer.

## Phantom Outbreaks

Several features of the Forum are designed to prevent phantom outbreaks caused by the power of suggestion. Visitors cannot implicate any particular food item or exposure. Second, visitors enter data before being given the opportunity to view output that might influence their own reports. (While visitors may modify their responses on subsequent visits to the Forum, very few do.) Third, each visitor only views output for food sources, foods, and other risk factors they have already indicated in earlier data entry screens. Fourth, food stores, restaurants, and other food sources are identified to Forum visitors only by abbreviations, which may not be specific to particular establishments. Abbreviations are sufficient to identify suspicious clusters, but only health departments see the entire names of commercial establishments. Finally, suspicious clusters must be investigated by the local health department to determine if clusters are due to foodborne outbreaks, chance, confounding, pranks, or normal changes in diet.

## Pilot Test

The Forum is being pilot tested in the tri-county area of Clinton, Eaton, and Ingham counties, which make up the Greater Lansing, Michigan area. The Forum was implemented in November 2002, involving three local health departments: the Barry-Eaton District Health Department, the Ingham County Health Department, and the Mid-Michigan District Health Department. For comparison purposes, we evaluated previous foodborne illness reports from the population of these counties for the years 2000 and 2001 (*4*–*6*).

### Publicity

The target population was 450,000 residents living in the three pilot counties. The percentage of this population that has Internet access is unknown, but an estimated 51.2% of all Michigan households had Internet access in 2001 (*7*). This percentage does not include persons who may have access at work, school, or public libraries.

Advertisements were published five times a week in the area’s daily newspaper (The Lansing State Journal). Fliers and brochures were distributed to 450 Lansing-area physicians in October 2002 with a letter explaining the project. Local television channels featured the project on various newscasts. In addition, three newspaper articles about the project appeared in the daily newspaper (*8*–*10*), two articles appeared in the university’s independent student newspaper (*11*,*12*), and one article ran in a smaller weekly paper (a subsidiary of the area’s largest newspaper) and a local township paper (*13*,*14*). Articles have been printed in various health departments’ newsletters and other university-related publications.

A student employee worked 2 days per week visiting private physicians’ offices, urgent care offices, emergency rooms, and pharmacies to distribute fliers and brochures and to ask clinic nurses to recommend the Forum to patients with suspected foodborne illness. The Forum has been described at local grand-rounds meetings for internal medicine, emergency medicine, pediatrics, and family practice.

Publicity given to this project may have influenced the results. The Forum was well advertised in the three-county pilot area. Reports could have increased as a result of advertising, regardless of mode of reporting. However, one local health department involved in the pilot project stated that the number of traditional reports had not increased since the outset of the RUsick2 Forum.

## Illness Reporting

Table 2 depicts the Michigan Department of Agriculture data collected for 2000 and 2001 by year, month, and county. From these data, we predicted that approximately 22 reports would have been expected during the comparable months of 2002 and 2003. The RUsick2 data are displayed by month and county in Table 3 and show that 93 reports were obtained with the Web-based system.

**Table 2:** Foodborne disease reports in Michigan by county, month, and year^a^

County	Y	Jan	Feb	Nov	Dec	
Ingham	2000	9	4	4	3	
	2001	1	2	0	4	
Clinton	2000	0	0	0	0	
	2001	0	0	0	0	
Eaton	2000	1	2	3	1	
	2001	4	4	1	2	
Total		15	12	8	10	
No. wk/m		4.42	4.00	4.29	4.42	
2000–2001 average/wk		1.70	1.50	0.93	1.13	

^a^Source: Michigan Department of Agriculture, unpub. data.

**Table 3:** RUsick2 visits by county, month, and year

County	Y	Nov	Dec	Jan	Feb
Ingham	2002	15	13	--	--
	2003	--	--	25	9
Clinton	2002	0	1	--	--
	2003	--	--	1	3
Eaton	2002	7	7	--	--
	2003	--	--	12	3
Total		22	21	38	12
No. wk/m		4.29	4.42	4.42	4.00
Average/wk		5.13	4.75	8.60	3.00

From November 2002 (the time the Forum was implemented) until February 2003, a total of 93 reports to the RUsick2 Forum reached at least the entry level of reporting (which begins by identifying foods consumed). In the first 17 weeks of the program, an average of 5.37 cases were reported each week. Based on the previous years’ reports, we calculated an expected number of 1.31 cases per week; hence, the ratio of reported cases to expected reports (5.37/1.31) was 4.10.

**Figure 3:**
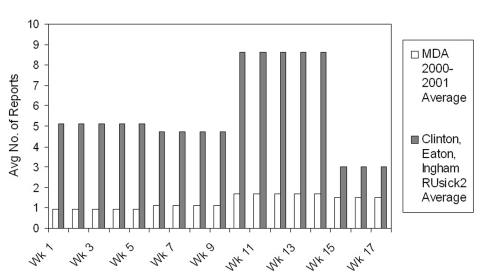
Comparison of foodborne disease reports from Ingham, Eaton, and Clinton Counties before and after implementing the RUsick2 Forum; MDA, Michigan Department of Agriculture.

Figure 3 shows the weekly average of foodborne complaints reported to the Forum from the three pilot counties during the first 17 weeks of operation. Also shown on this graph is the weekly average number of foodborne disease complaints reported to the state of Michigan during the corresponding months of January, February, November, and December 2000–2001. Approximately 22 reports would have been expected during the 17-week period, based on reports from previous years. However, 93 reports were received during the first 17 weeks of operation, more than a fourfold increase in reporting.

Local health departments monitored reports to the Forum and contacted reporting persons by telephone or email to verify the accuracy of the report and authenticate the complaint. Contacts usually required a few minutes. Reports delivered from the Forum to local health departments were treated as traditional reports. The Forum identified two foodborne outbreaks that would likely not have been identified. One fictitious report was identified and rejected.

## Future Development

Similar to other Web-based forums, RUsick2 allows persons with a common health problem to examine one another’s information. In an attempt to determine if they share any common exposures, visitors can view risk factors such as food histories, food sources, and other exposures entered by previous visitors. The Rusick2 Forum acts as a “front end” to existing surveillance by increasing reporting and identifying suspicious clusters that warrant a full investigation.

The Forum has recently been adapted for national usage so that the technical aspects of data entry and information retrieval will function identically for residents of all states. The publicity campaign conducted in the pilot counties will be too expensive to reproduce on a national level, so the Forum will only gain national prominence if local health departments and consumer advocacy groups use and publicize the Web site. The input screens of this program are easily modifiable or removable. For example, the symptoms screen can be altered to include symptoms specific to a non-foodborne disease, or the food history screens can be deleted entirely. Thus, this program could be adapted for use in other disease outbreaks.

## References

[1] Mead PS, Slutsker L, Dietz V, McCaig LF, Bresee JS, Shapiro C, Food-related illness and death in the United States. Emerg Infect Dis. 1999;5:607–25. 10.3201/eid0505.990502 10511517 PMC2627714

[2] Food practices and diarrheal diseases: *vox populi*. CD Summary [newsletter on the Internet]. Center for Disease Prevention and Epidemiology, Oregon Health Division. 1998 Nov 24; 47(24). Available from: http://www.dhs.state.or.us/publichealth/cdsummary/1998/ohd4724.pdf

[3] Centers for Disease Control and Prevention. Standard foodborne disease outbreak case questionnaire. 2002 Mar [cited 2003 Mar 21]. Available from: http://www.cdc.gov/foodborneoutbreaks/standard_questionnaire.htm

[4] U.S. Census Bureau. State and county quickfacts: Clinton county. 2002 Sept [cited 2002 Nov 15]. Available from: http://quickfacts.census.gov/qfd/states/26/26037.html

[5] U.S. Census Bureau. State and county quickfacts: Eaton county. 2002 Sept [cited 2002 Nov 15]. Available from: http://quickfacts.census.gov/qfd/states/26/26045.html

[6] U.S. Census Bureau. State and county quickfacts: Ingham county. 2002 Sept [cited 2002 Nov 15]. Available from: http://quickfacts.census.gov/qfd/states/26/26065.html

[7] U.S. Department of Commerce. Economics and Statistics Administration: National Telecommunications and Information Administration. A nation online: How Americans are expanding their use of the internet [online]. 2002 Feb [cited 2002 Dec 2]. Available from: http://www.ntia.doc.gov/ntiahome/dn/nation_online.pdf

[8] Terlep S. MSU develops unique method to track food-poisoning cases: system will let people report sickness online. The Lansing State Journal 2002 May 20; Sect. A:1, A:5.

[9] Harrison S. Web site lets users report food poisoning. The Lansing State Journal 2002 Nov 4; Sect. B:1.

[10] Trout S. More food ailments reported online. The Lansing State Journal 2003 Jan 5; Sect. B:1.

[11] Byrne K. Web site to aid food poisoning reporting. The State News 2002 May 22; 3.

[12] Korneffel S. Group sets up web site on contaminated foods. The State News 2002 Nov 26; 7.

[13] Miller M. Got the dry heaves? Tell us please. NOISE 2003 Feb 5.

[14] Michigan State University asks Lansing area: RUsick2? The Ingham County Community News 2003 Jan 12; 18.

